# Contribution of APCs to mucosal-associated invariant T cell activation in infectious disease and cancer

**DOI:** 10.1177/1753425918768695

**Published:** 2018-04-09

**Authors:** Muki Shehu Shey, Avuyonke Balfour, Katalin Andrea Wilkinson, Graeme Meintjes

**Affiliations:** 1 Wellcome Centre for Infectious Diseases Research in Africa, Institute of Infectious Diseases and Molecular Medicine, University of Cape Town, Observatory 7925, South Africa; 2 Department of Medicine, Faculty of Health Sciences University of Cape Town, Observatory 7925, South Africa; 3 The Francis Crick Institute, Midland Road, London, NW1 2AT

**Keywords:** Antigen presenting cells, cancer, infectious disease, IL-12, IL-18, MAIT cell activation, MR1

## Abstract

APCs such as monocytes and dendritic cells are among the first cells to recognize invading pathogens and initiate an immune response. The innate response can either eliminate the pathogen directly, or through presentation of Ags to T cells, which can help to clear the infection. Mucosal-associated invariant T (MAIT) cells are among the unconventional T cells whose activation does not involve the classical co-stimulation during Ag presentation. MAIT cells can be activated either via presentation of unconventional Ags (such as riboflavin metabolites) through the evolutionarily conserved major histocompatibility class I-like molecule, MR1, or directly by cytokines such as IL-12 and IL-18. Given that APCs produce cytokines and can express MR1, these cells can play an important role in both pathways of MAIT cell activation. In this review, we summarize evidence on the role of APCs in MAIT cell activation in infectious disease and cancer. A better understanding of the interactions between APCs and MAIT cells is important in further elucidating the role of MAIT cells in infectious diseases, which may facilitate the design of novel interventions such as vaccines.

## Introduction

Following a pathogenic infection, innate cells, especially APCs such as dendritic cells (DCs), monocytes/macrophages, and B cells recognize and initiate an immune response to eliminate the pathogen. The innate response can either eliminate the pathogen directly, or through presentation of Ags to T cells to activate the adaptive immune response that helps clear the infection.^1^ Unlike an innate response, the T cell response is typically delayed and develops several hours or days following infection and priming by innate cells.^2^ Several T cell subsets exist, including the conventional (CD4^+^ and CD8^+^) and the unconventional T cells or donor-unrestricted T cells (DURT) including gamma delta, NK T cells (NKT) and mucosal-associated invariant T (MAIT) cells.^3^ MAIT cells are T cells with innate-like functions and have recently gained much attention as important players in immunity to infectious diseases and in the pathogenesis of certain non-communicable diseases such as obesity, cancer, and diabetes. Unlike conventional T cells, which are activated solely through Ag presentation, MAIT cells can be activated either via Ag presentation through the evolutionarily conserved MHC class I (MHC-I)-like molecule, MR1, or directly by cytokines.^4^ Given that APCs produce cytokines and can express MR1 necessary for Ag presentation, these cells play an important role in both pathways of MAIT cell activation. In this review, we summarize what is known and highlight the knowledge gaps regarding the role of conventional APCs such as monocytes and DCs in the activation of MAIT cells in peripheral blood and the tissue environment.

## MAIT cells: phenotype, location and function

**Figure 1.**
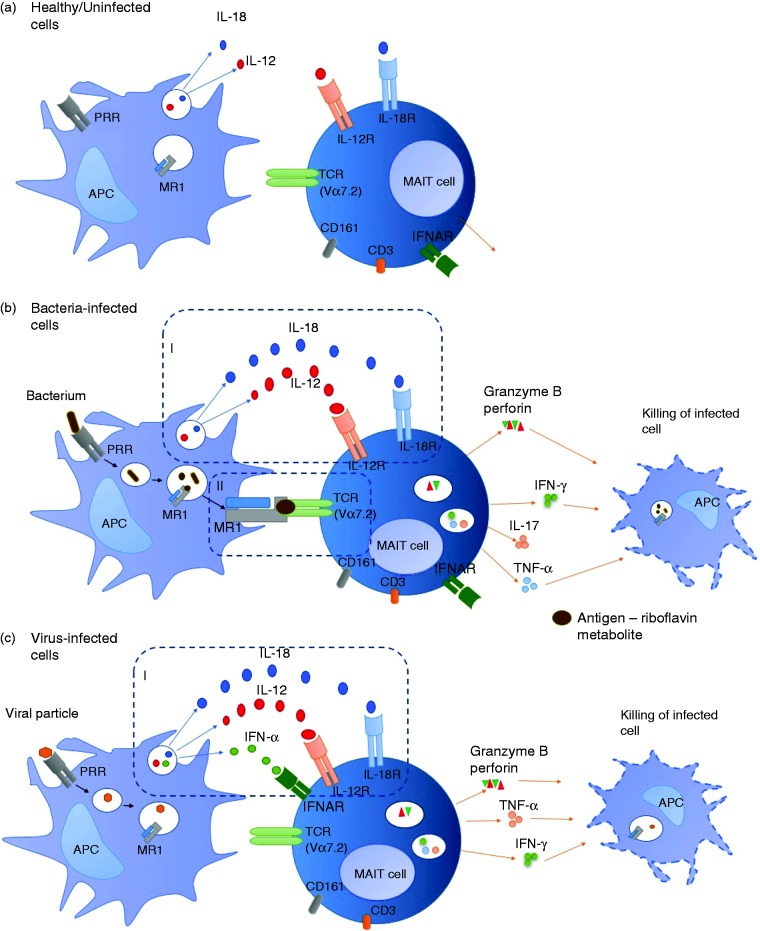
Interactions between APCs and MAIT cells in health and disease. (a) In the absence of infection, APCs may still produce cytokines as part of house-keeping processes but not enough to activate MAIT cells. (b) During bacterial infection, APCs produce large amounts of cytokines able to activate MAIT cells in an MR1-independent manner (I) or MAIT cells are activated through Ag presentation on MR1 (II). MAIT cells produce effector molecules such as IFN-γ, TNF-α and cytotoxic molecules required for killing and eliminating bacteria-infected cells. (c) Viral infection results in cytokine-dependent activation of MAIT cells (I). MAIT cells produce effector molecules such as IFN-γ, TNF-α and cytotoxic molecules required for killing and eliminating virus-infected cells.

MAIT cells were first described in 1999, by the sequencing of their TCRs in purified CD8α^+^-enriched or CD8α^+^-depleted peripheral blood lymphocytes in humans. Similar cells were found in mice and cattle.^5^ It is only in the last 15 yr that the function of these cells has been described as research on MAIT cells has expanded due to their recognized role in anti-microbial immunity (Figure 1).^6–8^ MAIT cells are described as evolutionarily conserved T cells with innate-like properties and a limited T-cell receptor repertoire.^5,9^ These cells are primarily CD8^+^ or CD4^–^CD8^–^ (double negative), and a smaller subset are CD4^+^.^10^ Human MAIT cells express CD161 at high levels with co-expression of the invariable T cell receptor Vα7.2 or TRAV1-2.^11,12^ Co-expression of CD161 and CD26 has also been used to define these cells in humans.^13^ Recently, ligand-loaded MR1 tetramers have been developed, and may be used for more consistent identification of MAIT cells.^14,15^ The MAIT TCR repertoire is quite diverse and heterogeneous. Although the TRAV1-2/TRAJ33 sequence is the most commonly used, other sequences, such as TRAJ20 and TRAJ12, are also used, depending on the pathogen.^16^

MAIT cells recognize vitamin derivatives and pyrimidines found in bacteria including *Escherichia coli*, Bacillus Calmette-Guerin (BCG), *Mycobacterium tuberculosis* (Mtb) and some fungi, which are presented through MR1 and thereby activate MAIT cells.^9,14^ The specific vitamin B metabolites serving as MR1-restricted ligands for MAIT cell activation include the non-activating folic acid metabolite, 6-formyl pterin (6-FP), and the highly potent riboflavin (vitamin B2) metabolite, reduced 6-hydroxymethyl-8-D-ribityllumazine (rRL-6-CH_2_OH).^17^ When activated, MAIT cells can proliferate, produce cytokines (including IFN-γ, TNF-α, IL-17) and express cytotoxic molecules including granzymes, granulysin and perforin.^10,18^ The expression of cytotoxic molecules confers to MAIT cells the ability to directly kill pathogen-infected cells through lysis or apoptosis of infected cells.^4,7,19^ Some evidence suggested site-dependent differences in MAIT cell function in response to bacterial stimulation with MAIT cells from the female genital tract producing more IL-17 and IL-22, and less IFN-γ and TNF-α compared with MAIT cells in peripheral blood.^20^ Even though MAIT cells can be activated through the TCR-dependent (MR1) or independent (cytokine) pathways, the relative contribution from each of these pathways is not well defined, and likely depends on the pathogen eliciting the response. TCR-dependent activation of MAIT cells has been reported to arise early during stimulation, is short-lived, while long-term activation of effector MAIT cells is dependent on cytokines (TCR-independent).^21,22^ The degree of activation of tissue MAIT cells is limited (reflected in lower production of cytokines), even though these cells exhibit more rapid activation (reflected in broad up-regulation of gene expression) than blood MAIT cells, suggesting that the restriction of memory MAIT cell activation by TCR-dependent pathway in tissues is necessary to avoid unwanted activation in the absence of infection.^21^ Compared to other T cell subsets, MAIT cells have been shown to display primarily an effector memory phenotype (CCR7^–^CD45RA^+^) upon activation and in patients with active TB.^23^

Recent reports suggest that, in contrast to their antimicrobial properties, MAIT cells can also induce immunopathology and immunosuppression in response to superantigens such as staphylococcal enterotoxin B (SEB).^24^ SEB induced an exaggerated and rapid cytokine production by MAIT cells compared to (non-MAIT) CD4^+^, CD8^+^, gamma-delta and invariant NK (iNK) T cells, resulting in up-regulation of programme death 1 (PD1), T cell immunoglobulin and mucin 3 (TIM3) and lymphocyte activation gene 3 (LAG-3), which rendered MAIT cells anergic to *Klebsiella pneumoniae* and *E. coli* stimulation. These MAIT cell responses to SEB were independent of MR1, but highly dependent on SEB-induced IL-12 and IL-18 production.^24^

## APCs: Monocytes, DCs and B cells – function, location, and activation during pathogenic infection

APCs are among the first cells to recognize invading pathogens and initiate an immune response.^25^ The major APCs are DCs, monocytes/macrophages and B cells. Three distinct DC subsets have been described, including plasmacytoid DCs (pDCs; CD14^–^CD123^+^CD11c^–^), myeloid DCs (mDCs; CD14^–^CD123^–^CD11c^+^), found in blood, and Langerhans cells (LCs; CD1a^+^ or langerin^+^; found in tissues), which differ in phenotypic and functional properties, including expression of different receptors for pathogen recognition and the type of cytokines produced.^26,27^ Monocytes in human blood have been subdivided into three subsets with different functions in inflammation: classical monocytes characterized by high level expression of CD14 and low expression of CD16 (CD14^++^CD16^–^), non-classical monocytes with medium level expression of CD14 and high expression of CD16 (CD14^+^CD16^++^), and intermediate monocytes, characterized by low expression of CD16 and medium to high expression of CD14 (CD14^+^CD16^+^ or CD14^++^CD16^+^).^28,29^ Although the best-known function of B-cells is the Ab production leading to the formation of immune complexes that will help the clearance of microbes, B-cells are also considered to be classical APCs that can also directly influence MAIT responses via Ag presentation and cytokine production.^30,31^ In addition, B cells and DCs also express lectin-like transcript-1 (LLT1), a ligand for CD161 used to identify MAIT cells.^32–34^ B cells are essential for the development and maintenance of MAIT cells in humans and mice.^35^

APCs recognize pathogens through PRRs of which TLRs are the most widely studied. These receptors recognize PAMPs derived from microbial pathogens or danger-associated molecular patterns (DAMPs, also known as alarmins) derived from stressed cells and tissue injury, to initiate an immune response. The type of PRR initially triggered may determine the outcome of an innate immune response. This initial innate response may dictate the subsequent type of adaptive immune response mounted in response to an infection. Activated APCs produce cytokines and up-regulate expression of HLA class I/II and co-stimulatory molecules (including CD86, CD80 and CD40).^36^ Upon phagocytosis of pathogens such as mycobacteria including Mtb, APCs may eliminate the pathogens by either direct killing (for example via degradation in the lysosome),^37^ and/or presenting Ags derived from these pathogens to activate T cells. Three signals are required to activate conventional T cells during Ag presentation: (a) binding of the Ag-MHC complex with the T cell receptor (Signal 1); (b) the binding of the co-stimulatory molecules (such as CD80, CD86) on APC with CD28 on T cells (Signal 2); and (c) the production of cytokines by APCs that act on T cells (Signal 3).^38^ Activated T cells including CD4^+^ and CD8^+^ cells produce cytokines such as IFN-γ, or cytotoxic molecules such as granzymes or perforin. IFN-γ can activate innate cells to kill the pathogen through the production of NO products, and cytotoxic molecules can directly kill infected cells.^39,40^

Among the cytokines produced by APCs with diverse immune functions are IL-12 and IL-18, which are involved in activation of T cells including MAIT cells (discussed below). IL-12 plays a major role in polarization of T cell immunity towards a T helper type 1 (Th1) phenotype during Ag presentation in pathogenic infections including HIV and TB.^41–43^ IL-12 has been shown to enhance CD4^+^ and CD8^+^ immune responses in HIV infection; and IL-12-mediated Ag-specific T cell proliferation was correlated with the stage of chronic HIV infection.^44^ Elevated levels of IL-18 have been described in patients with HIV infections possibly contributing to sustained immune activation in this group of patients.^45^ Experiments in animal models demonstrated the importance of IL-12 and IL-18 in immune responses to TB, with mice lacking these cytokines unable to control Mtb infection.^42,46^ Sustained IFN-γ immune responses necessary for the control of TB are often mediated by IL-12.^47^

## MAIT cell activation and regulation by APCs

Unlike conventional CD4^+^ and CD8^+^ T cells, MAIT cells can be activated in two different ways (Table 1); (a) activation through Ag presentation to MAIT cells on MR1 in a TCR-dependent manner; (b) direct activation in a TCR-independent manner by cytokines (such as IL-12, IL-15, IL-18) produced by pathogen-infected/activated innate cells. Different pathogens activate MAIT cells through one or both of the above-mentioned ways. Viruses activate MAIT cells through cytokines in a TCR-independent manner, as these pathogens lack the Ags in their metabolic pathways required to activate MAIT cells through MR1 Ag presentation.^48^ Unlike viruses, most bacteria can activate MAIT cells through Ag presentation and cytokines. DCs and monocytes infected with BCG can produce cytokines including IL-12 and IL-18,^49^ as well as degrade the bacteria to Ags that may be presented by MR1 to MAIT cells for activation.^4^ Several other signaling pathways are involved in regulation of MAIT cell activation including PD-1 and signaling via TLRs on innate cells that result in the production of the cytokines that activate MAIT cells.^23,50^ The relative contribution to MAIT cell activation from each of these pathways and Ag presentation to TCRs has not been defined.

**Table 1.** Summary of studies that directly evaluated the mechanisms of MAIT cell activation in different pathogenic infections and diseases.

Pathogen type or disease	Pathways of MAIT cell activation	Experimental system	Major findings	References
Mtb (TB)	MR1	PBMC-derived (CD8^+^ and CD8^–^CD4^–^) MAIT cells co-cultured with Mtb-infected A549 cell lines	Mtb-reactive MAIT cell clones were broadly reactive, enriched in lungs and detected Mtb-infected epithelial cells	^12^
	MR1	Co-culture of Mtb-infected DC or epithelial cells with MR1-restricted T cell clone (D426B1)	Mtb-infected lung epithelial cells efficiently stimulated IFN-γ release by MAIT cells	^51^
BCG	MR1 (regulated by PD-1 signaling)	PBMC stimulated with live BCG	MAIT cells from TB patients exhibited an increase (and a decrease) in IFN-γ production in response to BCG (and *E. coli*) respectively.	^23^
	MR1 and IL-12p40^a^	Knockout mice, *in vivo*	MAIT cells inhibited intracellular BCG growth in macrophages	^4^
	MR1^a^	MR1 tetramer in macaques	BCG vaccination induced MAIT cell activation at site of vaccination	^59^
*E. coli*	MR1 and IL-12/IL-18	PBMC cultured with cytokines and PBMC-derived Vα7.2^+^ cells co-cultured with *E. coli*-activated monocytes	MAIT cells were activated by *E. coli*-pulsed monocytes	^55,60^
	Mainly MR1	THP1 cells co-cultured with CD8^+^ MAIT cells	Licensed MAIT cells killed *E. coli*-infected cells via granzyme B.	^10^
	Mainly IL-12/IL-18 with MR1 contribution	PBMC-derived CD8^+^ (MAIT) cells	Both CD161^++^Vα7.2^+^ MAIT and CD161^++^Vα7.2^−^ non-MAIT CD8^+^ cells responded to cytokine stimulation	^22^
*Candida albicans*	MR1	PBMC-derived Vα7.2^+^ cells co-cultured with *C. albicans* -activated monocytes	MAIT cells responded to *C. albicans* and *E. coli* using distinct TCRs	^60^
Enteric bacteria (*E. coli strains, S*. typhi) *S*. typhi	MR1	B cell-line and primary B cells infected with enteric bacteria were co-cultured with primary MAIT cells in PBMC Healthy volunteers infected with *S*. typhi and followed up for 28 d for development of typhoid fever	MAIT cells were activated in an MR1 dependent manner to produce cytokines, and express CD107a/b and CD69 MAIT cell numbers decreased in volunteers who developed disease, with a simultaneous increase in MAIT cell cytokine production, activation and proliferation	^53^ ^61^
*H. pylori*	MR1	*H. pylori*-infected macrophages co-cultured with blood or gastric tissue-derived MAIT cells	MAIT cells were activated in an MR1 dependent manner resulting in production of cytokines, expression of CD107a and CD69, and killing of infected macrophages	^56^
HCV	IL-18 in synergy with IL-12, IL-15 and IFN-α/β	Co-culture of PBMC-derived CD8^+^ MAIT cells with HCV-infected macrophages	HCV-activated MAIT cells restricted HCV replication	^48^
	IL-18 in synergy with IL-12 and IFN-α	Culture of PBMC with IL-12/IL-18 or IL18/ IFN-α	MAIT cells frequencies were reduced in chronic HCV infection even after successful treatment.	^62^
DENV	IL-18 in synergy with IL-12, IL-15 and IFN-α/β	Co-culture of PBMC-derived CD8^+^ MAIT cells with DENV-infected DCs	MAIT cells were activated by DENV to produce IFN-γ and granzyme B	^48^
Influenza virus	IL-18 in synergy with IL-12 and IL-15	Co-culture of PBMC-derived CD8^+^ MAIT cells with influenza-infected macrophages	MAIT cells were activated by influenza virus to produce IFN-γ and granzyme B	^48^
	IL-18	PBMCs co-cultured with influenza-infected epithelial cells	MAIT cells were protective against influenza infection in IL-18 dependent manner	^54^
	IL-18	Co-culture of influenza-infected monocytes with A549 MAIT cell line	As above	^54^
Cancer	TCR dependent	Cultured PBMCs	MAIT cells infiltrated colorectal cancer tumors and were activated to cause cell cycle arrest reducing viability of tumor cells	^63^
	TCR dependent	Cultured colonic-derived cells	MAIT cells infiltrated colon tumors, displaying an activated memory phenotype with reduced IFN-γ production.	^64^

a Experiments in animal model. HCV: Hepatitis C virus; DENV: Dengue virus; Mtb: *Mycobacterium tuberculosis*; BCG: Bacillus Calmette-Guerin; *S*. typhi: *Salmonella enterica* serovar Typhi.

The involvement of MR1 in MAIT cell activation was demonstrated by blocking MR1 or using MR1-deficient mice to show that MAIT cell responses to bacterial stimulations are significantly reduced (Table 1).^4,12^ In most experiments to date, MAIT cell activation through MR1 or the MR1 expression has been evaluated in non-primary cells, MR1-transfected cell lines or epithelial cells.^12,51^ Compared to MR1-transfected cell lines, MR1 expression on primary innate cells is suggested to be transient and this may result in lower frequencies (compared to MR1-transfected cells) of activated cytokine-producing MAIT cells upon stimulation with bacteria.^50,52^ MR1 is localized in intracellular vesicles in primary APCs and relocates to the cell surface following binding of Ag or infection of cells.^51^ MR1 expression on B cells (cell line) and epithelial cells has been shown to be significantly up-regulated after infection with different species of enteric bacteria (*E. coli* strains and *Salmonella enterica* serovar typhi (*S*. typhi)).^53^

Few studies have reported the role of DCs, monocytes and B cells in activation of MAIT cells. When MAIT cells were co-cultured with DCs and macrophages infected with hepatitis C virus (HCV), Dengue virus (DENV) or influenza virus, there was an increase in MAIT cell activation resulting in the production of IFN-γ, TNF-α and granzyme B, and up-regulation of CD69 by MAIT cells.^48^ In that study, it was further shown that the replication of HCV in liver hepatocytes was restricted by activated MAIT cells. When co-cultured with monocytes that were activated through stimulation of TLR8 ligand (single-stranded RNA), MAIT cells were activated to produce IFN-γ and Granzyme B, and activation was higher than co-culturing MAIT cells with supernatants obtained from TLR8-stimulated monocytes.^21^ These findings suggest monocytes activate MAIT cells in both a contact-dependent (even in absence of Ag presentation on MR1) and -independent manner. MAIT cell were activated to a similar degree when co-cultured with influenza-infected monocytes or monocyte-derived macrophages, driven mostly by IL-18 cytokine.^48,54^ When MAIT cells were co-cultured with monocytes pulsed with fixed *E. coli*, these MAIT cells were also activated to produce cytokines and up-regulate activation markers in both MR1-dependent and independent manner.^55^ *Helicobacter pylori*-infected THP1 (monocyte)-derived macrophages were shown to activate MAIT cells to produce cytokines and express CD107a and CD69.^56^ Activation of MAIT cells also resulted in an increased in killing of the infected macrophages in an MR1-dependent manner. When co-cultured with *E. coli-* and *S*. typhi-infected B cells (both cell-line and primary B cells), MAIT cells became activated and produced cytokines, expressed CD107a/b and CD69 T cell activation marker in an MR1-dependent manner.^53^ Furthermore, B cell Ag presentation on MR1 to activate MAIT cells was also shown to be regulated by TLR9.^57^ In addition, activated MAIT cells were able to kill *E. coli-*exposed B cells in an MR1-dependent manner through granzyme A, B and perforin mechanisms.^10^ Studies evaluating the direct activation of MAIT cells, in an MR1-dependent or-independent manner, by APCs upon infection/stimulation by bacteria in an autologous system are lacking and this is required to further understand the role APCs play in the activation of MAIT cells *in vivo* in infectious disease.

Whereas APCs are required for activation of MAIT cells, MAIT cells have also been shown to play a role in the activation of APCs such as DCs. When peripheral blood-derived MAIT cells were co-cultured with immature monocyte–derived DCs, there was up-regulation of maturation markers (CD80, CD83, CD86 and PD-L1) on DCs and IL-12 production in a CD40 ligand-dependent manner, and this DC maturation was further enhanced in the presence of LPS, suggesting a regulatory role of MAIT cells.^58^

## Changes in MAIT cell numbers and function in disease states

MAIT cells can be found in blood, tissues and airways. These cells make up 1–10% of circulating T cells in peripheral blood, and up to 60% of T cells in liver and gastrointestinal tract.^9,15,65–67^ The frequencies of MAIT cells in peripheral blood is lower in patients with TB disease or HIV infection compared to healthy individuals without TB or HIV, respectively.^23,68^ Lower MAIT cell numbers in blood has also been described in patients with sepsis, cholera and human T-lymphotropic virus type 1 (HTLV-1) infections, as well as non-infectious conditions such as diabetes, cystic fibrosis, autoimmunity, cancer and obesity.^69–76^ In contrast, MAIT cell numbers have been shown to increase in the lungs of patients with pulmonary TB, suggesting a role in local immune responses to the infection.^12^ The reason for the decrease in MAIT cell numbers in the periphery in these conditions is not well understood. It has been suggested that the decrease in MAIT cell frequencies in blood could be due to redistribution to tissues, activation-induced cell death or down-regulation of receptors used to identify these cells, such as CD161.^8,12,77,78^ The recent development of the MR1 Ag-loaded tetramers may add clarity to the above conflicting findings by improved identification of MAIT cells in situations where CD161 is down-regulated, compromising detection by mAbs.^15^ Frequencies of MAIT cells have been shown to decrease in peripheral blood of individuals infected with *S*. typhi and *H. pylori* but their functional capacity (cytokine production, activation or proliferation) increased compared with uninfected individuals.^56,61^

There have been conflicting reports in HCV infection whether MAIT cells increase or decrease in liver compared to the periphery.^11,79^ Eberhard et al.^79^ described a decrease in MAIT cells (defined as CD4^–^CD3^+^CD161^+^Vα7.2^+^) frequencies in the liver, while Billerbeck et al.^11^ described an enrichment of MAIT cells (defined as CD3^+^CD8^+^CD161^+^) in the liver compared with blood. The differences in MAIT cell definition might contribute to the conflicting findings.

Even though it is well described that MAIT cell frequencies in blood decrease in certain diseases, the function of these cells during infection and other diseases is not yet well described. Studies report conflicting findings regarding functional attributes (e.g. level of cytokine production) of MAIT cells in TB disease in blood and this may relate to pathogen used for *ex vivo* stimulation.^23,80^ A decrease in IFN-γ production by MAIT cells in blood was described in TB patients compared with healthy controls upon *E. coli* stimulation, and this was associated with an increase in PD-1 expression in these cells.^80^ Upon BCG stimulation, MAIT cells from TB patients exhibited an increase in IFN-γ and TNF-α production while *E. coli* stimulation in the same patients was associated with a decrease in cytokine production.^23^ Upon PD-1 blockade followed by BCG or *E. coli* stimulation, MAIT cells from TB patients exhibited an increase in IFN-γ production in comparison to no PD-1 blockade.^23^ Increased production of cytokines, granzyme B and up-regulation of the activation markers (HLA-DR and CD38) by MAIT cells has also been described in chronic HCV and DENV infection in an IL-18-dependent manner, despite the decrease in frequencies of MAIT cells.^48,79^ One study in HIV infection reported that despite an early depletion, MAIT cells remained highly activated (high expression of HLA-DR and CD38) and their functional capacity (IFN-γ production) was retained up to 2 yr after HIV seroconversion.^81^

## MAIT cell activation and function in infectious diseases

The activation of MAIT cells by bacterial and viral pathogens suggest that these cells could play a role in preventing the establishment of infection after exposure or preventing progression from infection to disease, in the case of TB, for example. BCG vaccination in non-human primates resulted in activation of MAIT cells in blood 14- to 28-d post vaccination but conventional non-MAIT CD8^+^ T cells were not activated.^59^ MAIT cells were also found to proliferate more, became more activated and expressed higher granzyme B at the site of vaccination (chest) with BCG compared to distal site (thigh). When macaques were infected with Mtb, MAIT cells proliferated more in blood, but the level of activation was similar to that of conventional non-MAIT CD8^+^ cells.

Kwon et al.^80^ and Jiang et al.^82^ showed that MAIT cell responses to bacteria in pulmonary TB patients were functionally defective compared to healthy controls, but similar in people with non-tuberculous mycobacteria suggesting the inhibitory effect of mycobacterial disease on MAIT cell function. A polymorphism on MR1 has been linked to altered MR1 expression, susceptibility to TB and death from meningeal TB.^83^ The minor allele genotype of the polymorphism was associated with increased susceptibility to meningeal TB and higher MR1 expression, suggesting over-activation of MAIT cells may promote inflammation, which may paradoxically be detrimental by resulting in bacterial dissemination from lungs to extra pulmonary sites including the central nervous system.^83^ A possible increase in cerebral inflammation could also be a contributing factor. More studies are required to evaluate the association of MAIT cells with TB at presentation and disease outcome. Several other human studies (discussed above) have described the activation, functional and numerical changes in MAIT cells in patients with TB and other bacterial/viral diseases.^12,75,80,82^ So far, no human studies have associated MAIT cell function or numbers with clinical outcomes of disease or infection. MAIT cells have been shown to accumulate in the lungs of mice infected with *S*. typhi and this accumulation was dependent on MR1 and the size of bacterial inoculum.^84^ In addition, differentiation of monocytes to DCs in the lungs following *Francisella tularensis* infection and subsequent recruitment of CD4^+^ T cells to the lung was mediated by MAIT cells.^85^ IL-12p40-, MR1- or MAIT cell-deficient mice lacked the ability to control BCG or *F. tularensis* infections and quickly succumbed to low doses of BCG infection.^4,86^

In a human challenge model, individuals were infected with high (10^4^) and low (10^3^) doses of *S.* typhi and followed up for up to 28 d for the development of typhoid fever.^61^ In individuals who developed the disease (compared with those that did not) there was an increase in proliferation (using Ki67), expression of CD38 and HLA-DR (activation markers), CD57 (exhaustion marker), and caspase 3 (apoptosis marker) on MAIT cells and these changes occurred 48–96 h after disease onset. In individuals infected with *H. pylori*, compared with uninfected individuals, MAIT cell numbers were lower in peripheral blood but not the gastric mucosa.^56^ However, MAIT cells in both peripheral blood and gastric mucosa were activated by *H. pylori*-infected macrophages in an MR1-dependent manner to express cytokines, CD107a and CD69, and these MAIT cells were also able to directly induce killing of the infected macrophages.^56^

Despite effective treatment (leading to significant improvement in CD4 counts and control of viraemia) for HIV for up to 4 yr, MAIT cell frequencies were still comparable to pre-treatment levels, but the function of these cells was partially restored.^68,77,78^ Similarly, the decrease in MAIT cell frequencies in HCV infection did not recover following successful 24-wk HCV therapy.^62,79^ Other evidence suggests that the decrease in MAIT cell numbers correlate with the type of bacterial disease and severity,^70,75,80^ and numbers may rise upon effective antibacterial treatment; patients whose MAIT cell numbers failed to rise upon treatment were more susceptible to further hospital acquired infections.^70^

## MAIT cell activation and function in cancer

MAIT cells are also affected by cancer and other non-communicable diseases such as obesity, diabetes, autoimmunity, and asthma, and may play a role in the pathogenesis of these conditions.^63,64,69–72,74–76,87^ Changes in MAIT cell numbers and function have been reported in different forms of cancer.^63,64,76,87^ It was observed that the frequencies of circulating MAIT cells tend to decrease in patients with intestinal cancers such as colorectal cancer and gastric cancer, as well as lung cancers, compared to MAIT cells in healthy controls.^76^ On the contrary, for non-mucosal associated cancers such as liver and thyroid cancer, the frequencies and numbers of MAIT cells in circulation were similar to those of healthy controls, and higher than those of mucosal associated cancer patients.^76^ Despite a decrease in circulating MAIT cells, there was accumulation of MAIT cells in tumors.^63,87^ High frequencies of MAIT cells were observed in colorectal tumors compared to healthy colons, while frequencies of other T cell subsets were similar in these tissues.^63,87^

Cytokine production by MAIT cells either decreased or remained similar in cancer patients compared to healthy individuals. One study observed no significant difference in cytokine production by MAIT cells between cancer patients and healthy controls in responses to phorbol myristate acetate (PMA) and ionomycin,^76^ while other studies observed a lower IFN-γ and TNF-α production in tumor-associated MAIT cells compared to MAIT cells in unaffected mucosa.^63,64^ IL-17 production by MAIT cells was either higher in cancer patients compared with healthy controls,^76^ or similar.^64^

MAIT cells may have a direct cytotoxic effect on cancer cells. To assess their functions, Ling et al.^63^ and Won et al.^76^ co-cultured MAIT cells using HCT116 (human colon cancer cell line) and K562 (a human erythroleukemic cell line) cells. Activated MAIT cells produced cytokines (TNF-α, IFN-γ and IL-17), degranulated (increased CD107a expression) with up-regulation of cytotoxic markers (perforin and granzymes B) during co-culture, and had the capacity to cause cell cycle arrest of HCT116 at the G2/M cycle in a cell-cell contact-dependent manner, resulting in reduced viability of HCT116 and K562 cells.^63,76^

The infiltration of tumors by MAIT cells and the resulting cytokine and cytotoxic marker expression suggest that these cells play a role in anti-tumor immunity such as modeling the cytokine network and affecting the balance between tumor suppressing and promoting cytokines, and may also have a role in the development or the regulation of tumors, although their specific function and the interaction with innate cells in these processes still needs to be defined. An association of MAIT cell numbers with outcome in cancer has been suggested: an increase in MAIT cell infiltration of tumors correlated with poor survival in patients with colorectal cancer.^87^ However, the changes in MAIT cell function and numbers upon cancer treatment has not been described.

## Conclusion and future perspectives

We have summarized here the existing (albeit limited) evidence that APCs can activate MAIT cells in both an MR1-dependent and independent manner. These studies have used mostly MR1 transfected cell lines, epithelial cells or heterologous systems where APCs are activated separately followed by co-culture of either infected cells or cell culture supernatants with MAIT cells. Studies assessing MAIT cell activation by APCs in an autologous system are lacking. Such studies are needed to more precisely define the interactions of APCs with MAIT cells resulting in MAIT cell activation, as well as the relative contribution of the MR1-dependent and independent pathways of MAIT cell activation. Also, research is needed to understand how the magnitude of MAIT cell activation is related to the quality and quantity of innate cell activation and the magnitude of cytokine production by innate cells.

Despite several studies reporting the decrease in frequencies of MAIT cells in peripheral blood and some tissues in HIV, TB, other viral and non-communicable diseases, further studies are required to delineate the specific functions of MAIT cells compared with conventional T cells in these diseases and how they associate with presentation and outcome. To the best of our knowledge, no human studies have so far shown an association between alteration of MAIT cell function or numbers/frequencies with clinical outcomes such as protection from infection or development of disease. Even though MAIT cells seem to play a role in immune responses that may prevent infection or progression to disease, more studies are required to demonstrate this. A better understanding of the activation and function of MAIT cells in infectious and non-communicable diseases including HIV, TB, and cancer may lead to more targeted interventions to prevent or treat these diseases, such as the development of mucosal or systemic vaccines that specifically activate MAIT cells.
